# Theory for Cavity-Modified Ground-State Reactivities via Electron–Photon Interactions

**DOI:** 10.1021/acs.jpca.3c01421

**Published:** 2023-07-27

**Authors:** Arkajit Mandal, Michael A. D. Taylor, Pengfei Huo

**Affiliations:** † Department of Chemistry, University of Rochester, 120 Trustee Road, Rochester, New York 14627, United States; ‡ Department of Chemistry, Columbia University, New York, New York 10027, United States; § Institute of Optics, Hajim School of Engineering, University of Rochester, Rochester, New York 14627, United States

## Abstract

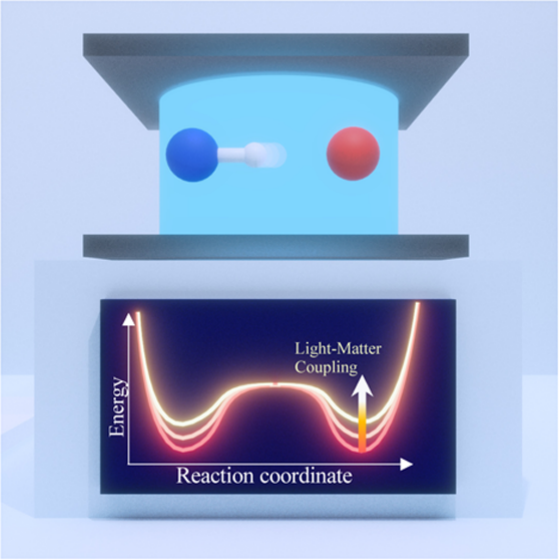

We provide a simple and intuitive theory to explain how coupling a molecule to an optical cavity can modify ground-state chemical reactivity by exploiting intrinsic quantum behaviors of light–matter interactions. Using the recently developed polarized Fock states representation, we demonstrate that the change of the ground-state potential is achieved due to the scaling of diabatic electronic couplings with the overlap of the polarized Fock states. Our theory predicts that for a proton-transfer model system, the ground-state barrier height can be modified through light–matter interactions when the cavity frequency is in the electronic excitation range. Our simple theory explains several recent computational investigations that discovered the same effect. We further demonstrate that under the deep strong coupling limit of the light and matter, the polaritonic ground and first excited eigenstates become the Mulliken–Hush diabatic states, which are the eigenstates of the dipole operator. This work provides a simple but powerful theoretical framework to understand how strong coupling between the molecule and the cavity can modify ground-state reactivities.

## Introduction

Coupling molecules to a quantized radiation field inside an optical cavity creates a set of photon-matter hybrid states, so-called polaritons. These polariton states hybridize the curvatures from both the ground and the excited electronic states and have shown great promise to alter the photochemistry of molecules.^1−4^ Unlike traditional photochemistry, which uses light as an energy source, polariton chemistry uses quantized photons as active chemical catalysts to significantly change the shape of the potential energy surface (PES) in molecular systems and thus open up new possibilities to tune and control chemical reactions.^5−8^

Theoretical investigations play a vital role in understanding the fundamental limits and the basic principle of new chemical reactivities achieved by polariton chemistry.^7−10^ In particular, the molecule–cavity interactions have often been described as the following dipole gauge Hamiltonian
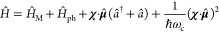
1where  is the molecular Hamiltonian, *Ĥ*_ph_ is the photon field Hamiltonian, *â*^†^ and *â* are the raising and lowering operators of the cavity field, and ***μ*^** is the dipole operator of the molecule. Further, ***χ*** = χ**ê**, where χ describes the coupling strength and **ê** indicates the cavity field polarization direction. This Hamiltonian predicts that there will be couplings between the photon-dressed states, for example, |*g*,1⟩ ≡ |*g*⟩⊗|1⟩ (the ground electronic state with 1 photon) and |*e*,0⟩ ≡ |*e*⟩⊗|0⟩ (the excited electronic state with 0 photons), through ⟨*g*,1| ***μ*^**(*â*^†^+ *â*)|*e*, 0⟩= ***μ***_*ge*_⟨1|(*â*^†^+ *â*)|0⟩. When the energy of these two states is very close, the |*g*,1⟩ and |*e*,0⟩ states hybridize, leading to the formation of polariton states. Using such a simple Hamiltonian, it has been shown that the presence of the cavity can suppress^11^ or enhance^8,12^ photo-isomerizations,^11−13^ increase charge transfer rates by orders of magnitude,^14−16^ modify potential energy landscapes even with no photon in the cavity,^8,11,17−19^ enhance electron-phonon coupling strength,^20^ accelerate singlet fission kinetics,^21^ remotely control chemical reactions,^22^ enhance excitation energy transfer processes,^16,23,24^ and create new polariton induced conical intersections.^8,18,25,26^ All of these emerging features of polariton chemistry demonstrate a great promise to control and tune chemical reactivities, as the cavity quantum electrodynamics (QED) processes take advantage of the quantum nature of light and its interaction with the molecular system.

Recently, several theoretical works have demonstrated that the ground state of a molecular system can be significantly modified by coupling to a cavity photon mode with a photon frequency in the electronic excitation range.^27−34^ In particular, ref (35) demonstrates that the aminopropenal proton-transfer reaction barrier can be modified by coupling the system with the cavity. Note that the cavity frequency in these studies is chosen to be in the range of electronic excitation (in terms of energy, a couple of eV), thus different than the recently explored vibrational strong coupling (VSC) regime^36,37^ (in terms of energy, on the order of 100 meV). These modifications are caused by indirect couplings between different photon-dressed states due to the presence of both transition and permanent dipoles.^38^ For example, the |*g*,0⟩ state couples with |*g*,1⟩ through ⟨*g*,1|***μ*^**(*â*^†^ + *â*)|*g*, 1⟩ = ***μ***_*gg*_⟨1|(*â*^†^ + *â*)|0⟩ and |*g*,1⟩ couples to the |*e*,0⟩ through ***μ***_*ge*_⟨1|(*â*^†^ + *â*)|0⟩. As such, the |*g*,0⟩ and the |*e*,0⟩ states are indirectly coupled to each other (through the light–matter interactions), and the ground-state properties can also be significantly influenced under the strong light–matter interaction coupling strength. Further, the dipole self-energy (DSE) term (see eq 1, the last term) could also significantly influence the ground-state properties and reactivities, as demonstrated in ref (35). This is because the DSE operator can be expressed as  where |α⟩∈{|*g*⟩,|*e*_1_⟩···|*e*_*i*_⟩···}. One can clearly see that the DSE term is able to connect electronic adiabatic states far away in energy. However, an intuitive and simple understanding of cavity modification of the molecular ground state is still missing. As such, the cavity-modified chemical reactivity in the ground state is an interesting new direction but not well understood due to those indirect effects as we mentioned above. We note that the existence of the DSE in the light–matter Hamiltonian has been a subject of debate in recent works with conflicting formulations of the light–matter Hamiltonian.^13,39−46^ It has been argued in ref (39) that even for pure electrostatic interactions in plasmonic cavities, the Hamiltonian should have a dipole self-energy (DSE) like a quadratic term. As such, we explicitly include the DSE term in this work.

In this paper, we provide a new theory and mechanism to explain how the ground-state chemical reactivity for a molecular system can be modified by coupling to the cavity with electronic excitation frequency. Our theory is based on the recently developed polarized Fock states (PFS) representation, which uses shifted cavity Fock states associated with each specific electronic state, similar to the widely used polaron transformation,^47^ Merryfield transformation,^48,49^ or Lang–Fersov transformation.^50,51^ These PFSs are nonorthogonal to each other but provide a compact description of the photonic Hilbert space. Using this polarized Fock states representation, we demonstrate that the change of the ground-state potential is achieved due to the scaling of diabatic electronic couplings with the overlap of the polarized Fock states. As the light–matter coupling increases, the diabatic electronic coupling is effectively reduced, resulting in a ground polaritonic potential energy surface that resembles the diabatic potential (associated with the dipole operator’s eigenstate).

Our theory predicts that for a proton-coupled electron transfer model system (Shin–Metiu model^52^), the ground-state barrier height can be modified through light–matter interactions when the cavity frequency is in the electronic excitation range. This novel change in the ground-state chemical reactivity is achieved through strong electronic coupling as opposed to the more common use of vibrational strong coupling for modifying the ground state.^36,37,53−55^ Our simple theory explains several recent computational investigations that discovered the same effect for proton-transfer systems^35,56,57^ and has the potential to add valuable insights into many more ground-state polariton studies.^21,27−33,58−69^ We further demonstrate that under the deep strong coupling limit of the light and matter, the polaritonic ground and first excited eigenstates become the Mulliken–Hush (MH) diabatic states, which are the eigenstates of the dipole operator. This work provides a simple but powerful theoretical framework to understand how strong coupling between molecule and cavity can modify ground-state reactivities.

## Theoretical Framework

### Theory of Polarized Fock States

We start by introducing the Pauli–Fierz (PF) nonrelativistic QED Hamiltonian^70−72^ to describe the light–matter interaction. The PF Hamiltonian can bederived^15,71,73^ by applying the Power–Zienau–Woolley (PZW) Gauge transformation^74,75^ and a unitary phase transformation^73^ on the minimal-coupling Hamiltonian in the Coulomb gauge (i.e., the “p·A” Hamiltonian) under the long-wavelength limit. A derivation is provided in the Appendix. For a molecule coupled to a single-photon mode inside an optical cavity, the PF Hamiltonian is
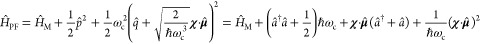
2which is eq 1 in the Introduction section. In the last line of eq 2, *Ĥ*_M_ represents the molecular Hamiltonian, the second term  represents the Hamiltonian of the vacuum photon field inside the cavity with the frequency ω_c_, and the third term describes the light–matter interaction in the electric-dipole “d·E” form,^75^ with  characterizing the light–matter coupling vector (or alternatively the electric field vector) oriented in the direction of polarization unit vector **ê**,  as the quantization volume for the photon field, and ε_0_ as the permittivity inside the cavity. Note that the parameters used in this work correspond to quantization volumes ranging from ≈13 to 530 nm^3^, which while challenging to achieve experimentally is well within the theoretically estimated limits as discussed in ref (76) and is much larger than the volumes used (≈0.2 nm^3^) in recent theoretical works.^35,69,77^ Further, *â*^†^ and *â* are the photon creation and annihilation operator,  and  are the photonic coordinate and momentum operator, respectively.

The total dipole operator of the entire molecule is
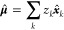
3where *z*_*k*_ is the charge for the *k*_th_ charged particle. Throughout this study, we assume that **ê** aligns with the direction of ***μ*^**. The last term is the dipole self-energy (DSE), which describes how the polarization of the matter acts back on the photon field.^9^ The PF Hamiltonian *Ĥ*_PF_ (eq 2) is what we expressed in eq 1 of the Introduction.

The matter Hamiltonian is expressed as follows
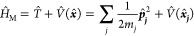
4where *j* is the index of the *j*_th_ charged particle (including all electrons and nuclei), with the corresponding mass, *m*_*j*_, and canonical momentum, **p̂**_*j*_ = −*i*ℏ**∇**_*j*_. We denote electronic coordinate with **r̂**, and nuclear coordinate with **R̂**, and use **x̂**_*j*_ ∈ {**r̂**_*j*_, **R̂**_*j*_} to represent either the electron or nucleus, with **x̂** being the coordinate operator for all charged particles. Further, **T̂** = **T̂**_**R**_ + **T̂**_**r**_ is the kinetic energy operator for all charged particles, where **T̂**_**R**_ and **T̂**_**r**_ represent the kinetic energy operators for nuclei and electrons, respectively. Further, *V̂*(**x̂**) is the potential operator that describes the Coulombic interactions among the electrons and nuclei. The electronic Hamiltonian is often defined as
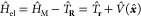
5which includes the kinetic energy of electrons, electron-electron interactions, electron-nuclear interactions, and nuclear–nuclear interactions. The essential task of the electronic structure community is focused on solving the eigenstates of *Ĥ*_el_ at a particular nuclear configuration **R** as follows
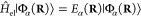
6where *E*_α_(**R**) is commonly referred to as the α_th_ potential energy surface (PES) or adiabatic energy, and |Φ_α_(**R**)⟩ is commonly referred to as the α_th_ adiabatic electronic state. The matrix elements of the total dipole operators can be obtained using the adiabatic states as
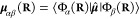
7For α ≠ β, ***μ***_αβ_(**R**) is referred to as the transition dipole between state |Φ_α_⟩ and |Φ_β_⟩, while ***μ***_αα_(**R**) is commonly referred to as the permanent dipole for state |Φ_α_⟩.

In a similar sense of defining the electronic Hamiltonian and corresponding eigenvalue equation for the matter, one can define the polaritonic Hamiltonian^9,30,62^ as *Ĥ*_pl_ ≡ *Ĥ*_PF_ – **T̂**_**R**_, which includes all operators of molecules and cavity, except for the nuclear kinetic energy operator **T̂**_**R**_. As such, the polariton state is defined as the eigenstate of *Ĥ*_pl_ through the following eigenvalue problem
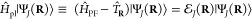
8where *Ĥ*_pl_ ≡ *Ĥ*_PF_ – **T̂**_**R**_ is the polariton Hamiltonian, |Ψ_*J*_(**R**)⟩ is the polariton eigenstate, and  is the polariton potential energy surface. As can be seen clearly, both |Ψ_*J*_(**R**)⟩ and  parametrically depend on the nuclear configuration **R**. We denote |Ψ_0_(**R**)⟩ as the ground state of *Ĥ*_pl_.

Next, we briefly introduce the idea of the Polarized Fock states, whereas the details for this theoretical development can be found in ref (38). In particular, we use the Mulliken–Hush (MH) diabatic representation to express the molecular Hamiltonian and the PF QED Hamiltonian. These Mulliken–Hush diabatic states {|μ_*i*_⟩} are the dipole operator’s eigenstates, such that
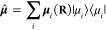
9where ***μ***_*i*_ is an eigen-dipole value associated with state |μ_*i*_⟩. These |μ_*i*_⟩ states can be obtained from the direct diagonalization of the ***μ*^** matrix in the adiabatic representation (with the matrix element expressed in eq 7). A good example of ***μ***_*i*_ could be the ionic and covalent dipole for a diatomic molecule, whereas the ***μ***_αβ_ are the transition and permanent dipoles (see Figure 1 of ref (38)). It should be noted that these MH diabatic states are not strict diabats since they are not completely independent of the nuclear coordinates. However, for a given nuclear configuration, they can be used as a decent approximation of diabats. Note that the nonadiabatic couplings that arise due to nuclear position dependence of these MH states are relevant for dynamics, but they do not contribute to the potential energy surfaces discussed here.

**Figure 1:**
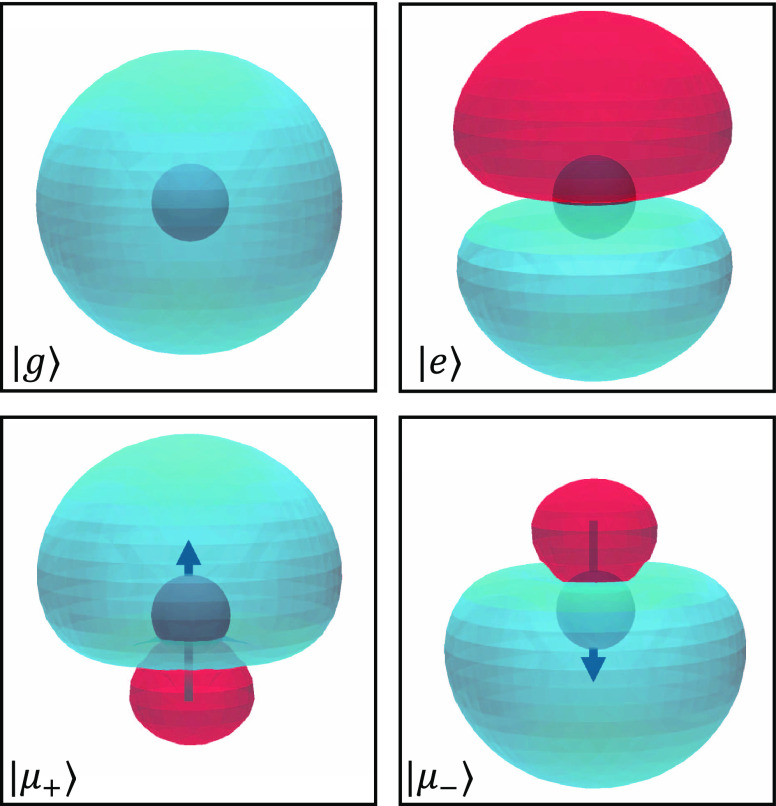
Visualization of an atomic system’s wave functions in its energy eigenstates (|*g*⟩ and |*e*⟩) and its first two MH diabatic states (|μ_+_⟩ and |μ_–_⟩).

Using the MH diabatic states, the matter Hamiltonian in eq 4 can be expressed as
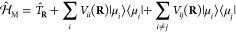
10where we have used the MH diabatic basis and *V*_*ii*_(**R**) represents the diabatic potentials, *V*_*ij*_(**R**) represents the diabatic coupling. This truncation of the matter Hilbert space is done by projecting the matter Hamiltonian to the lowest few eigenstates of *Ĥ*_el_ such that . Then, the MH diabatic states are formed by diagonalizing . We want to emphasize that the extent to which a matter subspace can be truncated accurately strongly depends on the system under consideration.

The PF Hamiltonian in eq 2 under a finite set of the |μ_*i*_⟩ basis is expressed as
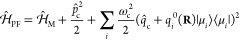
11where the diabatic state-specific shift *q*_*i*_^0^(**R**) is
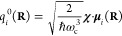
12

We notice that the photon field is described as a displaced Harmonic oscillator that is centered around −*q*_*i*_^0^(**R**). This displacement can be viewed as a polarization of the photon field due to the presence of the molecule–cavity coupling, such that the photon field corresponds to a non-zero (hence polarized) vector potential, in contrast to the vacuum photon field.

We introduce the polarized Fock state (PFS) |*n*_*i*_(**R**)⟩ ≡ |*n*_*i*_⟩ as the Fock state of a displaced Harmonic oscillator, with the displacement  specific to the diabatic state |μ_*i*_⟩ such that
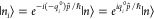
13where *n*_*i*_ = 0, 1, 2···, ∞ is the quantum number for the PFS. Compared to the vacuum’s Fock state |*n*⟩, this PFS depends on the diabatic state (or, more generally, the eigenstate of ***μ*^**) of the molecule, and the position of the nuclei (through the **R** dependence in ***μ***_*i*_(**R**)). Due to the electronic state-dependent nature of the polarization, the PFS associated with different electronic diabatic states become nonorthogonal,^38^ i.e., ⟨*n*_i_|*m*_j_⟩ ≠ δ_*ij*_δ_*nm*_.

To remove this shift of the photonic coordinate, a unitary transformation can be formed as
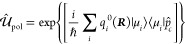
14where eq 14 is a shift operator of the form  such that it shifts an arbitrary operator *Ô*(*q̂*) by an amount *q*_0_, .

The polariton Hamiltonian under the polarized Fock state (PFS) representation can then be written as
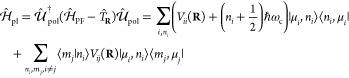
15The detailed expression of  can be found in ref (38). In eq 15, one can clearly see that as the light–matter coupling strength ***χ*** increases, the overlap function between the displaced harmonic oscillators decreases for different diabatic states. This is because the state-specific displacement, *q*_*i*_^0^, increases linearly with ***χ***, causing ⟨*m*_*j*_|*n*_*i*_⟩ → δ_*ij*_δ_*nm*_.

In the very strong coupling limit (e.g., an infinite light–matter coupling), the overlap ⟨*m*_*j*_|*n*_*i*_⟩ approaches zero, effectively eliminating all diabatic electronic couplings *V*_*ij*_(**R**). As such, the polariton Hamiltonian becomes , where the light and matter are effectively decoupled. This agrees with the recent theoretical analysis of atomic QED systems under the deep strong coupling limit.^78^ It also indicates that, as the light–matter coupling goes large, the polariton state
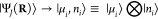
16meaning that the polariton state |Ψ_*J*_⟩ becomes the tensor product of Mulliken–Hush diabatic states with the PFS state. Further, the polariton potential energy surface becomes
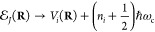
17which means the polariton state will assume the same potential curvature as the MH diabatic states *V*_*i*_(**R**) since the photonic energy  is **R**-independent.

Equations 16 and 17 are considered the main theoretical results in this work. Below, we will use both the atomic cavity QED example and a model molecular cavity QED system to demonstrate its predictive power.

#### Special Case of the Atomic Cavity QED

To give an illustrative and familiar example, let us consider the case of an atom
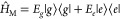
18where the transition dipole is ***μ****_eg_* = ⟨*e*|***μ*^**|*g*⟩. Note that the permanent dipoles are ***μ***_*ee*_ = ⟨*e*|***μ*^**|*e*⟩ = 0, ***μ***_*gg*_ = ⟨*g*|***μ*^**|*g*⟩ = 0. Thus, the dipole operator is expressed as
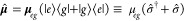
19by defining the creation operator σ̂^†^ ≡ |*e*⟩⟨*g*| and annihilation operator σ̂ ≡ |*g*⟩⟨*e*| of the electronic excitation. The atom-cavity PF Hamiltonian becomes . Dropping the DSE (the last term) leads to the Rabi model^79^
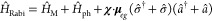
20where further dropping the counter-rotating terms σ̂^†^*â*^†^ and σ̂*â* leads to the well-known Jaynes–Cummings (JC) model,^80^ with the details provided in the Appendix.

For atomic systems where ***μ***_*ee*_ = ***μ***_*gg*_ = 0,the eigenstates of ***μ*^**, i.e., the MH diabatic states are
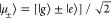
21which are commonly referred to as the qubit states^81−84^ in the atomic, molecular, and optical (AMO) physics community. Under this basis, the dipole operator becomes
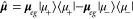
22with the eigenvalue ***μ***_±_ = ±***μ***_*eg*_. Figure 1 provides a visualization of the wave functions for an atomic system in both its energy eigenstates and its MH diabatic states.

Using these diabatic states, the atomic Hamiltonian is expressed as
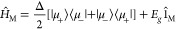
23where Δ = *E*_*e*_ – *E*_*g*_ and  is the identity operator for this matter model.

The PFS representation of this model then becomes
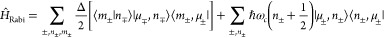
24since *V*_±_= 0, *V*_±,∓_ = Δ/2, and there are no nuclear DOFs. As discussed in ref (81), by approximating that ⟨*m*_±_|*n*_∓_⟩ ≈ 0 for *m* ≠ *n* (valid for large couplings), the Hamiltonian in eq 24 becomes block diagonal and the eigenenergies and eigenstates can be analytically obtained as follows^81,82^
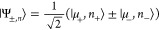
25a
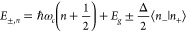
25bIt should be noted that this expression uses the notation from ref (81). These eigenstates should not be confused with the Jaynes–Cummings (JC) eigenstates (see eqs 50a and 50b) that are the exact eigenvectors of the JC Hamiltonian (see eq 49).

In the deep strong coupling limit, the overlap between the PFS ⟨*n*_–_|*n*_+_⟩ → 0. In this case, one can clearly see that the eigenstates |Ψ_±_,*n*⟩ become doubly degenerate with *E*_+,*n*_ = *E*_–,*n*_ and the MH diabatic states |μ_±_,*n*⟩ are the eigenstates of the light–matter Hamiltonian where the corresponding eigenenergies simplify to the energies of these MH states added to the energy of *n* photons.

## Models and Computational Details

In this paper, we use this Shin–Metiu three-level system^52^ to demonstrate two different regimes of ground-state modifications. To demonstrate the ground-state potential modification when coupling a molecule to the cavity, we use the Shin–Metiu^52^ single-electron proton-transfer model truncated to three matter levels for the matter Hamiltonian. This model consists of fixed donor and acceptor ions with a proton being transferred between them, yielding one nuclear coordinate. The matter truncation is done in the MH basis, creating a model three-level system with donor (D), acceptor (A), and covalent (C) diabatic states, such that {|μ_*i*_⟩}∈ {|D⟩, |A⟩, |C⟩}.

### Shin–Metiu Model

The Shin–Metiu model^52^ is a one-dimensional molecular system that describes a proton-coupled electron transfer reaction between a donor and an acceptor ion. The model consists of a transferring proton, an electron, and two fixed ions (a donor ion and an acceptor ion). The total Hamiltonian of the molecular system is *Ĥ*_M_ = T̂_*R*_ + *Ĥ*_el_, where *T̂*_*R*_ = *P̂*_*R*_^2^/2*m*_p_ is the kinetic energy of the transferring proton, with proton mass *m*_p_. In addition, *Ĥ*_el_ is the electronic Hamiltonian
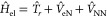
26where *T̂*_*r*_ = *p̂*_r_^2^/2*m*_e_ represents the kinetic energy operator of the electron with mass *m*_e_, *V̂*_eN_ describes the interaction between the electron and the three ions through the form of a modified Coulomb potential as follows
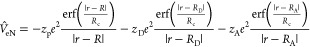
27where *r* is the position and *e* = 1 au is the fundamental charge, *R* is the position of the proton, while *R*_D_ and *R*_A_ are the positions of the static donor and acceptor ion, respectively. Further, *z*_p_, *z*_D_, and *z*_A_ represent the charge of the proton, donor ion, and acceptor ion, respectively. Additionally, *R*_c_ characterizes the strength of the modified coulomb interaction between the electron and the ions.

The nuclear–nuclear interaction potential *V*_NN_ describes the coulomb repulsion between the proton and the static ions as follows
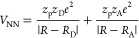
28

### Computational Details

In this work, we use the Fourier grid Hamiltonian (FGH) approach^85,86^ to solve the eigenvalue problem of all matter and polariton Hamiltonians. In particular, we use a total of *N* = 2000 grid basis {|*r*_*i*_⟩} to describe the electronic degrees of freedom *r* in the range [*R*_D_ – 10, *R*_A_ + 10] (with Δ*r* = 0.01 au), which allows us to solve the adiabatic states of the matter. The matrix elements of the electronic Hamiltonian *Ĥ*_el_ in this grid basis {|*r*_*i*_⟩} are given by
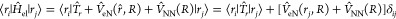
29where the ⟨*r*_*i*_ |*T̂*_*r*_ |*r*_*j*_⟩ is given analytically^85,86^ as follows
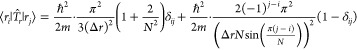
30

Directly diagonalizing the matrix of *Ĥ*_el_ at a given nuclear position *R* in this grid basis gives the accurate adiabatic electronic states, and in this work, we only focus on the lowest three adiabatic states
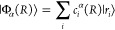
31where *a* ∈ {0,1,2}. Further, the matrix elements for dipole moment operator μ̂ = *R̂* + *r̂*, can be computed as
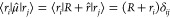
32After solving for the lowest three adiabatic PESs, we project this dipole operator to the truncated Hilbert space of these adiabatic states, such that , where . By defining  in this subspace, we can then find the matrix elements *V*_*i*_(**R**) and *V*_*ij*_(**R**) by transforming , where *V*_*i*_(**R**) and *V*_*ij*_(**R**) are the diagonal and off-diagonal matrix elements, respectively, in this basis.

Additionally, the overlaps of the shifted Fock states, ⟨*m*_*j*_|*n*_*i*_⟩ are found using the relation^81^
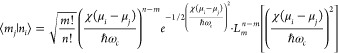
33for *m* < *n*, where *L*_*m*_^*n*–*m*^ is an associated Laguerre polynomial.

Using all of the above components, we can solve for the polariton potential energy surface  of the Hamiltonian  (eq 15).
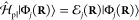
34The polaritonic PESs  can then be calculated by using the values of *V*_*i*_(**R**) and *V*_*ij*_(**R**) calculated from the pure matter system (through the above discrete variable representation method) and the PFS overlaps (eq 33), ⟨*m*_*j*_|*n*_*i*_⟩, and directly diagonalizing the matrix of . Note that identical results are obtained by solving eq 8 using the adiabatic-Fock basis |Φ_α_(*R*)⟩⊗|*n*⟩, where |Φ_α_(*R*)⟩ is the adiabatic states (see eq 6) and |*n*⟩ is the Fock state for the nonshifted field .

As expected from our theoretical analysis in eq 16, the polariton state approaches the tensor product of the Mulliken–Hush (MH) diabatic states and the PFS states as the light–matter coupling increases. The curvature of the polariton potential energy surface will also approach the MH diabatic states, as indicated in eq 17. In particular, for the Shin–Metiu three-level model system studied here, we consider three MH diabatic states, a donor state |D⟩ where the electron is localized on the donor atom, an acceptor state |A⟩ where the electron is localized on the acceptor atom, and an atomic state |C⟩ where the electron is localized on the transferring charged ion (proton). With a strong light–matter coupling strength, at a particular nuclear configuration *R*, the ground state of the light–matter hybrid will approach one of these diabatic states depending on which of the diabatic energies *V*_*ii*_(*R*) = ⟨*i*|*Ĥ*_el_|*i*⟩ with *i* ∈ {|D⟩, |A⟩, |C⟩} is the lowest.

## Results and Discussion

In this work, we consider two particular sets of parameters, which we refer to as model I and model II. The parameters in the molecular Hamiltonian *Ĥ*_M_ are tabulated in Table 1. In model I, when strongly coupled to the cavity, the ground polariton potential will approach the diabatic state |C⟩; for model II, the ground polariton potential approaches the acceptor and donor diabatic states |A⟩ and |D⟩, depending on the nuclear configuration. This will modify the reaction barrier on the ground state. In both cases, the molecular model is coupled to a highly off-resonant cavity with ω_c_ = 3 eV (thus operating outside the vibrational strong coupling regime^42,53,87^), and the number of Fock states was treated as a convergence parameter (converged with at most 50 Fock states for model I and 20 Fock states for model II).

**Table 1:** Parameters Used in the Molecular Hamiltonian *Ĥ*_M_ for Both Model I (Figure 2) and Model II (Figure 3)

parameter	model I	model II
*z*_p_, *z*_D_, *z*_A_	1 (unitless)	1 (unitless)
*R*_D_	–5.0 (Å)	–4.0 (Å)
*R*_A_	5.0 (Å)	4.0 (Å)
*R*_c_	1.0 (Å)	1.5 (Å)
*R*_n_	1.0 (Å)	1.0 (Å)
*m*_p_	1836 (au)	1836 (au)
*m*_e_	1 (au)	1 (au)

Additionally, it should be noted that for both models, this paper considers a single molecule strongly coupled to a cavity. Experimentally this has not been achieved with Fabry–Pérot (FP) cavities. However, recent exciting progress both experimentally^88^ and theoretically^76^ in plasmonic cavities demonstrating strong coupling at the few emitter levels provides hope for realizing these effects in the future. The effects described in this work can also be observed when the light–matter coupling is weaker by simply having a steady-state photon population, say *n* ≈ 10^1^–10^2^ (not enough photons to reach the Floquet limit^89^ where *n* → ∞). This is because the “diabatic” coupling terms are normalized by ⟨*n*_*j*_|*m*_*i*_⟩ terms which decay faster when *n*_*j*_ and *m*_*j*_ are large. Thus it may be possible to drastically modify the diabatic couplings at weaker light–matter couplings by compensating via many photons. Analogous results have also been derived for Floquet engineering of materials,^89,90^ where the coupling between Floquet blocks decays as the light intensity increases. For molecular systems, strong fields also induce many problems that do not exist in the single-photon limit since the molecule can then absorb many photons from the field and be excited, ionized, dissociated, etc.

We emphasize that for plasmonic cavities, in addition to the transverse fields, there are direct Coulomb interactions between the atoms of the plasmonic nanoparticles (or surface) and the molecule in question. The degree to which the electrostatic interactions or the coupling to the transverse field is important will depend on the distance between the molecules and the metal surface (or nanoparticle) surface and how fast the evanescent field decays.

**Figure 2:**
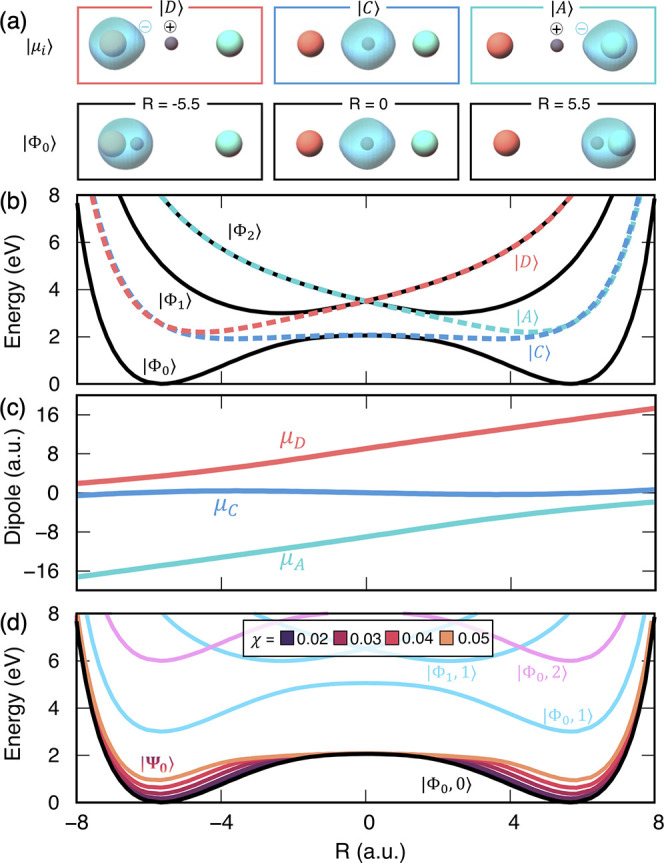
Model I: Shin–Metiu model for the hydrogen atom transfer reaction. (a) Visualization of electron charge density for the lowest energy diabatic states at three different nuclear positions and the ground-state adiabatic states at the same positions. (b) Plot of the PESs for the adiabatic states ({|Φ⟩}) and the diabatic states ({|D⟩, |A⟩, |C⟩}). (c) Dipole eigenvalues for each diabatic state as a function of *R*. (d) Polaritonic ground-state PES, |Ψ_0_⟩(*R*), displayed for various coupling strengths.

Figure 2 presents the results of coupling model I to the cavity. The diabatic states |D⟩, |C⟩, and |A⟩ in this model are depicted in the top panel of Figure 2a, whereas the ground adiabatic electronic states |Φ_0_(*R*)⟩ at three nuclear configurations are depicted below the diabatic states. In this model, the covalent diabatic state is lower in energy than the donor and acceptor diabatic states, as shown in Figure 2b (dashed lines). As a result, the character of the ground adiabatic state is dominated by the covalent diabatic state |C⟩, especially near the top of the potential barrier (where *R* ≈ 0). In this case, the proton and the electron are transferred simultaneously from the donor ion to the acceptor ion, making this process an adiabatic hydrogen atom transfer reaction. This is schematically illustrated in Figure 2a, where the adiabatic (bottom panels) and diabatic (top panels) ground-state electron distributions are illustrated at the donor (*R* = −5.5), barrier (*R* = 0), and acceptor (*R* = 5.5) nuclear configurations. This shows how the electron follows the proton along the proton-transfer coordinate. In Figure 2b, the adiabatic and diabatic potential energy surfaces are plotted. It can be seen that the two wells in the adiabatic ground-state potential energy surface come from the coupling between the donor/acceptor diabats |D⟩ and |A⟩ with the covalent diabatic state |C⟩ through the diabatic couplings *V*_DC_(*R*) and *V*_AC_(*R*).

Figure 2c depicts the permanent dipole moments of the three MH diabats as a function of *R*. Note that the transition dipoles among the MH diabats are zero by definition (see eq 9). As expected, the permanent dipole of the covalent state |C⟩ is flat as the electron density sticks to the transferring proton. On the other hand, the permanent dipoles of the |D⟩ and |A⟩ states are linear with respect to the position of the transferring proton as in these states the electron density is localized on the donor/acceptor atom such that the permanent dipole is approximately μ_D/A_(*R*) ≈ *R* – *R*_D/A_.

Figure 2d depicts the potential energy surface for the photon-dressed adiabatic states |Φ_α_,*n*⟩, as well as the polariton potential associated with the ground state |Ψ_0_⟩. Near the barrier region (*R* ≈ 0), the ground adiabatic state coincides with the covalent diabatic state |C⟩, where the ground-state potential is nearly identical to MH diabatic potential *V*_C_(*R*) for *R* ≈ 0. Thus, for this Shin–Metiu three-level system, even in the infinite light–matter coupling limit, the ground-state potential near the barrier region will not be modified as it is already very close to the MH diabatic state. In contrast, the ground-state potential can be expected to get significantly modified near the donor and acceptor wells as the ground state is formed by hybridizing the |D⟩ or |A⟩ with the |C⟩ states through a diabatic coupling. Increasing light–matter coupling effectively decreases the diabatic coupling ⟨0_C_|0_D_⟩·*V*_DC_ due to the decreasing ⟨0_C_|0_D_⟩ (with a similar effect for ⟨0_C_|0_A_⟩ ·*V*_AC_). Thus, it will bring the ground-state potential closer to the MH diabats’ potential energy surface, as suggested in eq 17. This means that near ground-state potential minima (for *R* ≈ −5.5 and 5.5), the ground-state polariton potential will be pushed upward as it approaches the MH diabats lying above, thus effectively reducing the ground-state barrier height.

Another interesting feature of this ground-state modification shown here is that it is not sensitive to the cavity frequency ω_c_ as long as the photon frequency is not much smaller than the electronic energy gaps. This can be seen by analyzing eq 33, where the ⟨0_*i*_ |0_*j*_⟩ overlap is a function of χ/ω_c_ (note that χ ∝ ω_c_ such that χ/ω_c_ is ω_c_-independent), which is photon-frequency-independent for Fabry–Pérot (FP) cavities. This is because χ explicitly scales as , where  is the cavity quantization volume. For a fixed mirror size in an FP cavity, , making χ ∝ω_c_. As such, the overlap ⟨0_*i*_ |0_*j*_⟩ is photon-frequency-independent, and the ground-state modification also remains independent of photon frequency as long as the photon frequency is not much smaller than the electronic energy gaps. For plasmonic cavities, the quantization volume is not as trivial to calculate, as such *A*_0_ is no longer frequency-independent.

However, when ℏω_c_ ≪ (*E*_*e*_ – *E*_*g*_), in addition to considering the light–matter coupling in terms of ⟨0_*i*_|0_*j*_⟩·*V*_*ij*_, the couplings ⟨0_*i*_|*n*_*j*_⟩ ·*V*_*ij*_ also become relevant because *E*_*e*_ – *E*_*g*_ ≈ *E*_*e*_ – *E*_*g*_ + *n*_*j*_ℏω_c_. Under this circumstance, the ground-state modification would become frequency dependent as smaller cavity frequencies bring more Fock states becoming energetically relevant. On the other hand, when photon frequency is comparable to or larger than electronic transition, the ground-state modification can be mainly understood by considering the ⟨0_*i*_|0_*j*_⟩*V*_*ij*_ term, and then the cavity modification becomes photon frequency-independent.

Model I, presented in Figure 2, is particularly salient in the context of recent computational work that investigates molecular hydrogen atom transfer reaction landscapes when coupling a molecule strongly to a cavity.^91^ In ref (91), Pavošević et al. model the proton-transfer reaction of a malonaldehyde molecule in the strong coupling regime. They found that when using self-consistent QED electronic structure methods (such as QED Hartree–Fock and QED coupled cluster level of theory), one observes a decrease in the barrier height of the proton-transfer reaction with strong electronic coupling. Our model through the PFS theory (eq 17) provides an intuitive explanation for this effect.

**Figure 3:**
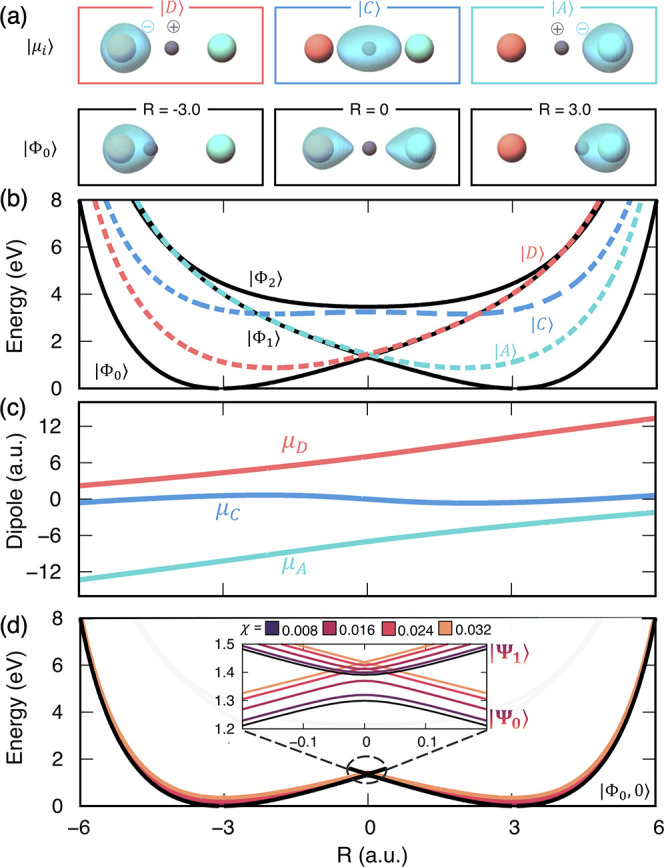
Model II: Shin–Metiu three-level proton-transfer model. (a) Visualization of electron charge density for the energy diabats at three different positions and the ground-state adiabat at the same positions. (b) Plot of the PESs for the adiabatic states ({|Φ⟩}) and the diabatic states ({|D⟩, |A⟩, |C⟩}). (c) Dipole eigenvalues for each diabatic state as a function of *R*. (d) Polaritonic ground-state PES, |Ψ⟩, and first excited state, |Ψ⟩, displayed for many coupling strengths.

Figure 3 presents a different kind of modification of the ground-state polariton potential. The parameters for model II are picked such that the covalent state |C⟩ lies energetically higher than the |D⟩ and |A⟩ states. Thus, the ground adiabatic state is largely composed of |D⟩ and |A⟩ states hybridized through the diabatic coupling *V*_DA_. As shown in Figure 3a, the electron density no longer follows along with the proton, especially in the middle panels of Figure 3a for *R* ≈ 0, in contrast to those in Figure 2. Instead, the charge density tunnels from the donor to the acceptor ion as the ground adiabatic state sharply changes its character from |D⟩ to the |A⟩ state near *R* ≈ 0. This model can be regarded as an example of a nonadiabatic proton-coupled electron transfer (PCET) reaction. Note that the permanent dipoles presented in Figure 3c are visually similar to the permanent dipoles shown in Figure 2c for model I, as the underlying nature of the MH diabats |D⟩, |A⟩, and |C⟩ is the same in both models.

The nonadiabatic transition from the donor to the acceptor state can be modulated by coupling to a cavity, following the same principles using PFS discussed above. As discussed before, the diabatic coupling *V*_DA_·⟨0_D_|0_A_⟩ will be reduced, which modulates the nonadiabatic transition near the barrier at *R* = 0. In particular, the splitting at the avoided crossing decreases with an increase in the light–matter coupling strength.^38^ This is illustrated in the inset in Figure 3d, which shows that the splitting gradually decreases with an increase in χ. Near the barrier, the lowest two polariton states approach the acceptor and donor diabatic states. In the limit of infinite light–matter coupling, the avoided crossing between the lowest two PESs becomes a conical intersection as the donor and acceptor states become decoupled.

These two models demonstrate different forms of ground-state modifications, but all are due to the same origin of the light–matter interactions that effectively scale down the diabatic couplings. In model I, the effective barrier height reduces significantly as the coupling increases. In model II, the avoided crossing between the first two adiabats decreases, approaching a conical intersection. In both cases, the fundamental mechanism is the same: the diabatic couplings between MH diabats decrease due to the decreasing overlap integral of two PFS, and the polariton potential energy surfaces approach the MH diabatic potential (eq 16) with increasing light–matter coupling. Under the infinite light–matter coupling limit, the polariton states reduce to the MH diabats tensor produced with PFSs (eq 17). The scaling of the diabatic couplings provides a new strategy for changing the ground-state chemical reactivity through electron–photon coupling in contrast to the operating principle of VSC, where cavity radiation can modify chemical reactivity through kinetics and dynamical effects.^42,53,92^ That said, this strategy is limited, especially in the case of degenerate eigenvalues of the dipole operator (in a suitable truncated electronic subspace) such that the photon field associated with these two electronic states are displaced to the same extent, and the overlaps ⟨*m*_*i*_|*n*_*j*_⟩ = δ_*mn*_ regardless of the light–matter coupling strength (where *i* and *j* label the degenerated MH diabatic states). As a result, the diabatic couplings between such dipole-degenerate states remain unmodified under such circumstances.

## Conclusions

Using the polarized Fock state (PFS) representation, we provide a simple theory to explain how strong electronic coupling can modify the ground-state polariton potential. We show that when coupled to the cavity, the diabatic couplings between Mulliken–Hush diabat states (which are the eigenstates of the electronic dipole operator) are modified. Using this theoretical framework, an increasing light–matter coupling will lead to a decrease of the diabatic coupling. In the infinite coupling limit, as the diabatic couplings go to zero, the MH diabats tensor product with the polarized Fock states become the eigenstates of the light–matter Hamiltonian (see eq 16). Consequently, as the coupling strength increases, the ground-state polariton PES approaches the lowest energy matter Mulliken–Hush diabatic state (see eq 17). This simple but intuitive theory explains several recently observed ground-state modifications due to strong light–matter interactions.^91^

In this paper, we demonstrate with three-level Shin–Metiu model systems that this modification of the ground-state either leads to a decrease of the relative barrier height (for model I in Figure 2) or a decrease of the avoided crossing between ground and excited states (for model II in Figure 3). This novel change in the ground-state chemical reactivity is achieved through strong electronic coupling as opposed to the more common use of vibrational strong coupling for modifying the ground-state chemical reactivity. This work provides a simple but powerful theoretical framework to understand how strong coupling between molecule and cavity can modify ground-state reactivities.

## Appendix: Derivation of the Pauli–Fierz QED Hamiltonians

We provide a brief derivation of the Pauli–Fierz QED Hamiltonian. The cavity photon field Hamiltonian under the single-mode assumption is expressed as

35

where ω_c_ is the frequency of the mode in the cavity, *â*^†^ and *â* are the photonic creation and annihilation operators, and  and  are the photonic coordinate and momentum operators, respectively. Choosing the Coulomb gauge, **∇**·**Â** = 0, the vector potential becomes purely transverse such that **Â** = **Â**_⊥_. Under the long-wavelength approximation,

36

where , with  as the quantization volume inside the cavity, ε_0_ as the permittivity, and **ê** as the unit vector of the field polarization.

The minimal-coupling QED Hamiltonian in the Coulomb gauge (the p·A form) is expressed as

37

where **p̂**_*j*_ = −*i*ℏ**∇**_*j*_ is the canonical momentum operator. We further introduce the Power–Zienau–Woolley (PZW) gauge transformation operator^74,75^ as

38

The PZW transformation operator can also be expressed as . Recall that a momentum boost operator  boosts *p̂* by the amount of *p*_0_, such that *Û*_p_ Ô(*p̂*) *Û*_p_^†^ = Ô(*p̂* + *p*_0_). Hence, *Û* simultaneously boosts the photonic momentum *p̂*_c_ by  and the matter momentum **P̂**_*j*_ by *z*_*j*_**Â**. Using *Û*^†^ to boost the matter momentum, one can show that

39

hence, *Ĥ*_C_ can be mathematically obtained^93^ by a momentum boost with the amount of −*z*_*j*_**Â** for **P̂**_*j*_ and then adding *Ĥ*_ph_.

The QED Hamiltonian under the dipole gauge (the d·E form^74,94^) can be obtained by performing the PZW transformation on *Ĥ*_C_ as follows

40

where we have used eq 39 to express *Ĥ*_C_, and the last three terms of the above equation are the results of *ÛĤ*_ph_*Û*^†^. Using *q̂*_c_ and *p̂*_c_, one can instead show that

41

because the PZW operator boosts the photonic momentum *p̂*_c_ by . Term  is commonly referred to as the dipole self-energy (DSE).

The Pauli–Fierz (PF) QED Hamiltonian^9,70,71^ can be obtained by using a unitary transformation  on *Ĥ*_D_. This is effectively a π/2 rotation in the phase space of the photonic DOF. To evaluate this, we use the following Baker–Campbell–Hausdorff (BCH) identity

42

Using the fundamental commutator [*â*^†^, *â*] = −1, we have [*â*^†^*â*, *â*] = *â*^†^[*â*, *â*] + [*â*^†^, *â*] *â* = −*â*. Denoting *Û*_ϕ_ = exp[*i*ϕ*â*^†^*â*] = *e*^–^*Â* (with ), *Â* = −*i*ϕ*â*^†^*â*. From the BCH identity, we have

43

Similarly, we have *e*^–*i*ϕ*â*^†^*â*^*â*^†^*e*^*i*ϕ*â*^†^*â*^ = *e*^–*i*ϕ^*â*^†^. Choosing  results in *Û*_ϕ_^†^*âÛ*_ϕ_ → *iâ* and *Û*_ϕ_^†^*â*^†^*Û*_ϕ_ → −*iâ*^†^. Using these results and applying *Û*_ϕ_ on *Ĥ*_D_, we have the PF Hamiltonian as follows

44

where the coupling constant is  as we used in eq 2. Note that we have used the fact that *Û*_ϕ_*Ĥ*_M_*Û*_ϕ_^†^ = *Ĥ*_M_, i.e., *Û*_ϕ_, is an identity for the matter DOFs. Hence, the role of *Û*_ϕ_ is to switch *p̂*_c_ and *q̂*_c_ since, for a photon field, they are interchangeable due to the pure harmonic nature of the quantized field. The PF Hamiltonian has the advantage of a pure real Hamiltonian, and the photonic DOF can be viewed^9,71^ and computationally treated^95,96^ as “nuclear coordinates”.

In quantum optics, a two-level atom coupled to a single model in an optical cavity is a well-studied subject. This leads to well-known model Hamiltonians, such as the Rabi model and the Jaynes–Cummings model. Since these two models are also widely used in recent investigations of polariton chemistry, here, we briefly derive them from the PF Hamiltonian.

We consider a molecule with two electronic states

45

and the transition dipole is ***μ***_*eg*_ = ⟨*e*|***μ*^**|*g*⟩. Note that the permanent dipoles in a molecule ***μ***_*ee*_ = ⟨*e*|***μ*^**|*e*⟩, ***μ***_*gg*_ = ⟨*g*|***μ*^**|*g*⟩ are not necessarily zero, as opposed to the atomic case where they are always zero. Hence, it is not always a good approximation to drop them.

The Rabi model assumes that one can drop the permanent dipoles, and leads to the dipole operator expression in the subspace  as follows

46

where we have defined the creation operator σ̂^†^ ≡ |*e*⟩⟨*g*| and annihilation operator σ̂ ≡ |*g*⟩⟨*e*| of the electronic excitation. The PF Hamiltonian (eq 44) in the subspace  thus becomes

47

Dropping the DSE (the last term) from eq 47 leads to the Rabi model

48

Dropping both the DSE and the counter-rotating terms σ̂^†^*â*^†^ and σ̂*â* leads to the well-known Jaynes–Cummings model^80^ as follows

49

The eigenstates of the JC model are analytically available, expressed as follows

50a

50b

where  is the mixing angle, and Δ*E* = *E*_*e*_ – *E*_*g*_ is the energy gap.

The limitations of these two models are thoroughly discussed in our recent work on Polariton-mediated charge transfer^73^ and polariton-mediated photodissociation dynamics of diatomic molecules.^38^

## References

[ref1] Hutchison J. A.; Schwartz T.; Genet C.; Devaux E.; Ebbesen T. W. Modifying Chemical Landscapes by Coupling to Vacuum Fields. Angew. Chem., Int. Ed. 2012, 51, 1592–1596. 10.1002/anie.201107033. 22234987

[ref2] Schwartz T.; Hutchison J. A.; Genet C.; Ebbesen T. W. Reversible Switching of Ultrastrong Light-Molecule Coupling. Phys. Rev. Lett. 2011, 106, 196405 10.1103/PhysRevLett.106.196405. 21668181

[ref3] Munkhbat B.; Wersall M.; Baranov D. G.; Antosiewicz T. J.; Shegai T. Suppression of Photo-Oxidation of Organic Chromophores by Strong Coupling to Plasmonic Nanoantennas. Sci. Adv. 2018, 4, eaas9552 10.1126/sciadv.aas9552. 29984306 PMC6035039

[ref4] Stranius K.; Hertzog M.; Börjesson K. Selective Manipulation of Electronically Excited States Through Strong Light-Matter Interactions. Nat. Commun. 2018, 9, 2273 10.1038/s41467-018-04736-1. 29891958 PMC5995866

[ref5] Ebbesen T. W. Hybrid Light-Matter States in a Molecular and Material Science Perspective. Acc. Chem. Res. 2016, 49, 2403–2412. 10.1021/acs.accounts.6b00295. 27779846

[ref6] Thomas A.; Lethuillier-Karl L.; Nagarajan K.; Vergauwe R.; George J.; Chervy T.; Shalabney T. C.; Shalabney A.; Devaux A.; Devaux E.; Genet E.; Genet C.; Moran C.; Moran J.; Ebbesen J. Tilting a Ground-State Reactivity Landscape by Vibrational Strong Coupling. Science 2019, 363, 615–619. 10.1126/science.aau7742. 30733414

[ref7] Kowalewski M.; Mukamel S. Manipulating Molecules with Quantum Light. Proc. Natl. Acad. Sci. U.S.A. 2017, 114, 3278–3280. 10.1073/pnas.1702160114. 28302671 PMC5380056

[ref8] Feist J.; Galego J.; Garcia-Vidal F. J. Polaritonic Chemistry with Organic Molecules. ACS Photonics 2018, 5, 205–216. 10.1021/acsphotonics.7b00680.

[ref9] Flick J.; Ruggenthaler M.; Appel H.; Rubio A. Atoms and Molecules in Cavities. Proc. Natl. Acad. Sci. U.S.A. 2017, 114, 3026 10.1073/pnas.1615509114. 28275094 PMC5373338

[ref10] Ribeiro R. F.; Martínez-Martínez L. A.; Du M.; Campos-Gonzalez-Angulo J.; Yuen-Zhou J. Polariton Chemistry: Controlling Molecular Dynamics with Optical Cavities. Chem. Sci. 2018, 9, 6325–6339. 10.1039/C8SC01043A. 30310561 PMC6115696

[ref11] Galego J.; Garcia-Vidal F. J.; Feist J. Suppressing Photochemical Reactions with Quantized Light Fields. Nat. Commun. 2016, 7, 13841 10.1038/ncomms13841. 27941754 PMC5159835

[ref12] Galego J.; Garcia-Vidal F. J.; Feist J. Many-Molecule Reaction Triggered by a Single Photon in Polaritonic Chemistry. Phys. Rev. Lett. 2017, 119, 136001 10.1103/PhysRevLett.119.136001. 29341675

[ref13] Galego J.; Garcia-Vidal F. J.; Feist J. Cavity-Induced Modifications of Molecular Structure in the Strong-Coupling Regime. Phys. Rev. X 2015, 5, 041022 10.1103/PhysRevX.5.041022.

[ref14] Herrera F.; Spano F. C. Cavity-Controlled Chemistry in Molecular Ensembles. Phys. Rev. Lett. 2016, 116, 238301 10.1103/PhysRevLett.116.238301. 27341263

[ref15] Semenov A.; Nitzan A. Electron Transfer in Confined Electromagnetic Fields. J. Chem. Phys. 2019, 150, 174122 10.1063/1.5095940. 31067889

[ref16] Schäfer C.; Ruggenthaler M.; Appel H.; Rubio A. Modification of Excitation and Charge Transfer in Cavity Quantum-Electrodynamical Chemistry. Proc. Natl. Acad. Sci. U.S.A. 2019, 116, 4883–4892. 10.1073/pnas.1814178116. 30733295 PMC6421448

[ref17] Kowalewski M.; Bennett K.; Mukamel S. Cavity Femtochemistry: Manipulating Nonadiabatic Dynamics at Avoided Crossings. J. Phys. Chem. Lett. 2016, 7, 2050–2054. 10.1021/acs.jpclett.6b00864. 27186666

[ref18] Kowalewski M.; Bennett K.; Mukamel S. Non-Adiabatic Dynamics of Molecules in Optical Cavities. J. Chem. Phys. 2016, 144, 054309 10.1063/1.4941053. 26851923

[ref19] Triana J. F.; Peláez D.; Sanz-Vicario J. L. Entangled Photonic-Nuclear Molecular Dynamics of LiF in Quantum Optical Cavities. J. Phys. Chem. A 2018, 122, 2266–2278. 10.1021/acs.jpca.7b11833. 29338227

[ref20] Sentef M. A.; Ruggenthaler M.; Rubio A. Cavity Quantum-Electrodynamical Polaritonically Enhanced Electron-Phonon Coupling and Its Influence on Superconductivity. Sci. Adv. 2018, 4, 041022 10.1126/sciadv.aau6969. PMC6269157 30515456

[ref21] Martínez-Martínez L. A.; Du M.; Ribeiro R. F.; Kéna-Cohen S.; Yuen-Zhou J. Polariton-Assisted Singlet Fission in Acene Aggregates. J. Phys. Chem. Lett. 2018, 9, 1951–1957. 10.1021/acs.jpclett.8b00008. 29551074

[ref22] Du M.; Ribeiro R. F.; Yuen-Zhou J. Remote Control of Chemistry in Optical Cavities. Chem 2019, 5, 1167–1181. 10.1016/j.chempr.2019.02.009.

[ref23] Du M.; Martínez-Martínez L. A.; Ribeiro R. F.; Hu Z.; Menon V. M.; Yuen-Zhou J. Theory for Polariton-Assisted Remote Energy Transfer. Chem. Sci. 2018, 9, 6659–6669. 10.1039/C8SC00171E. 30310599 PMC6115621

[ref24] Gonzalez-Ballestero C.; Feist J.; Moreno E.; Garcia-Vidal F. J. Harvesting Excitons Through Plasmonic Strong Coupling. Phys. Rev. B 2015, 92, 121402 10.1103/PhysRevB.92.121402.

[ref25] Bennett K.; Kowalewski M.; Mukamel S. Novel Photochemistry of Molecular Polaritons in Optical Cavities. Faraday Discuss. 2016, 194, 259–282. 10.1039/C6FD00095A. 27711849

[ref26] Szidarovszky T.; Halász G. J.; Császár A. G.; Cederbaum L. S.; Vibók A. Conical Intersections Induced by Quantum Light: Field-Dressed Spectra from the Weak to the Ultrastrong Coupling Regimes. J. Phys. Chem. Lett. 2018, 9, 6215–6223. 10.1021/acs.jpclett.8b02609. 30296095

[ref27] Flick J.; Ruggenthaler M.; Appel H.; Rubio A. Kohn–Sham approach to quantum electrodynamical density-functional theory: Exact time-dependent effective potentials in real space. Proc. Natl. Acad. Sci. U.S.A. 2015, 112, 15285–15290. 10.1073/pnas.1518224112. 26627715 PMC4687533

[ref28] Ruggenthaler M.; Flick J.; Pellegrini C.; Appel H.; Tokatly I. V.; Rubio A. Quantum-electrodynamical density-functional theory: Bridging quantum optics and electronic-structure theory. Phys. Rev. A 2014, 90, 012508 10.1103/PhysRevA.90.012508.

[ref29] Pellegrini C.; Flick J.; Tokatly I. V.; Appel H.; Rubio A. Optimized Effective Potential for Quantum Electrodynamical Time-Dependent Density Functional Theory. Phys. Rev. Lett. 2015, 115, 093001 10.1103/PhysRevLett.115.093001. 26371646

[ref30] Flick J.; Schäfer C.; Ruggenthaler M.; Appel H.; Rubio A. Ab Initio Optimized Effective Potentials for Real Molecules in Optical Cavities: Photon Contributions to the Molecular Ground State. ACS Photonics 2018, 5, 992–1005. 10.1021/acsphotonics.7b01279. 29594185 PMC5865078

[ref31] Haugland T. S.; Ronca E.; Kjonstad E. F.; Rubio A.; Koch H. Coupled Cluster Theory for Molecular Polaritons: Changing Ground and Excited States. Phys. Rev. X 2020, 10, 041043 10.1103/PhysRevX.10.041043.

[ref32] Mordovina U.; Bungey C.; Appel H.; Knowles P. J.; Rubio A.; Manby F. R. Polaritonic coupled-cluster theory. Phys. Rev. Res. 2020, 2, 023262 10.1103/PhysRevResearch.2.023262.

[ref33] DePrince A. E. Cavity-modulated ionization potentials and electron affinities from quantum electrodynamics coupled-cluster theory. J. Chem. Phys. 2022, 154, 094112 10.1063/5.0038748. 33685167

[ref34] Riso R. R.; Haugland T. S.; Ronca E.; Koch H. Molecular orbital theory in cavity QED environments. Nat. Commun. 2022, 13, 1368 10.1038/s41467-022-29003-2. 35292631 PMC8924263

[ref35] Pavošević F.; Hammes-Schiffer S.; Rubio A.; Flick J. Cavity-Modulated Proton Transfer Reactions. J. Am. Chem. Soc. 2022, 144, 4995–5002. 10.1021/jacs.1c13201. 35271261

[ref36] Thomas A.; Lethuillier-Karl L.; Nagarajan K.; Vergauwe R. M. A.; George J.; Chervy T.; Shalabney A.; Devaux E.; Genet C.; Moran J.; Ebbesen T. W. Tilting a ground-state reactivity landscape by vibrational strong coupling. Science 2019, 363, 615–619. 10.1126/science.aau7742. 30733414

[ref37] Thomas A.; George J.; Shalabney A.; Dryzhakov M.; Varma S. J.; Moran J.; Chervy T.; Zhong X.; Devaux E.; Genet C.; Hutchison J. A.; Ebbesen T. W. Ground-State Chemical Reactivity under Vibrational Coupling to the Vacuum Electromagnetic Field. Angew. Chem., Int. Ed. 2016, 55, 11462–11466. 10.1002/anie.201605504. PMC5113700 27529831

[ref38] Mandal A.; Vega S. M.; Huo P. Polarized Fock States and the Dynamical Casimir Effect in Molecular Cavity Quantum Electrodynamics. J. Phys. Chem. Lett. 2020, 11, 9215–9223. 10.1021/acs.jpclett.0c02399. 32991814

[ref39] Schäfer C.; Ruggenthaler M.; Rokaj V.; Rubio A. Relevance of the Quadratic Diamagnetic and Self-Polarization Terms in Cavity Quantum Electrodynamics. ACS Photonics 2020, 7, 975–990. 10.1021/acsphotonics.9b01649. 32322607 PMC7164385

[ref40] Feist J.; Fernández-Domínguez A. I.; García-Vidal F. J. Macroscopic QED for quantum nanophotonics: emitter-centered modes as a minimal basis for multiemitter problems. Nanophotonics 2020, 10, 477–489. 10.1515/nanoph-2020-0451.

[ref41] Semenov A.; Nitzan A. Electron transfer in confined electromagnetic fields. J. Chem. Phys. 2019, 150, 174122 10.1063/1.5095940. 31067889

[ref42] Mandal A.; Taylor M.; Weight B.; Koessler E.; Li X.; Huo P. Theoretical Advances in Polariton Chemistry and Molecular Cavity Quantum Electrodynamics. ChemRxiv 2022, g9lr7 10.26434/chemrxiv-2022-g9lr7. PMC10450711 37552606

[ref43] Taylor M. A. D.; Mandal A.; Huo P. Resolving ambiguities of the mode truncation in cavity quantum electrodynamics. Opt. Lett. 2022, 47, 1446 10.1364/OL.450228. 35290335

[ref44] Gustin C.; Franke S.; Hughes S. Gauge-invariant theory of truncated quantum light-matter interactions in arbitrary media. Phys. Rev. A 2023, 107, 013722 10.1103/PhysRevA.107.013722.

[ref45] Ryu C. J.; Na D.-Y.; Chew W. C. Matrix product states and numerical mode decomposition for the analysis of gauge-invariant cavity quantum electrodynamics. Phys. Rev. A 2023, 107, 063707 10.1103/PhysRevA.107.063707.

[ref46] Mandal A.; Xu D.; Mahajan A.; Lee J.; Delor M.; Reichman D. R. Microscopic Theory of Multimode Polariton Dispersion in Multilayered Materials. Nano Lett. 2023, 23, 4082–4089. 10.1021/acs.nanolett.3c01017. 37103998

[ref47] Nitzan A. Chemical Dynamics in Condensed Phases: Relaxation, Transfer and Reactions in Condensed Molecular Systems; Oxford University Press: Oxford, U.K., 2006.

[ref48] Merrifield R. E. Theory of the Vibrational Structure of Molecular Exciton States. J. Chem. Phys. 1964, 40, 445–450. 10.1063/1.1725135.

[ref49] Zhao Y.; Brown D. W.; Lindenberg K. Variational energy band theory for polarons: Mapping polaron structure with the Merrifield method. J. Chem. Phys. 1997, 106, 5622–5630. 10.1063/1.473598.

[ref50] Hohenadler M.; von der Linden W. Polarons in Advanced Materials; Springer: Netherlands, 2007; pp 463–502.

[ref51] Lang I.; Firsov Y. A. Kinetic theory of semiconductors with low mobility. Sov. Phys. JETP 1963, 16, 1301.

[ref52] Shin S.; Metiu H. Nonadiabatic Effects on the Charge Transfer Rate Constant: A Numerical Study of a Simple Model System. J. Chem. Phys. 1995, 102, 9285–9295. 10.1063/1.468795.

[ref53] Li X.; Mandal A.; Huo P. Cavity frequency-dependent theory for vibrational polariton chemistry. Nat. Commun. 2021, 12, 1315 10.1038/s41467-021-21610-9. 33637720 PMC7910560

[ref54] Li X.; Mandal A.; Huo P. Theory of Mode-Selective Chemistry through Polaritonic Vibrational Strong Coupling. J. Phys. Chem. Lett. 2021, 12, 6974–6982. 10.1021/acs.jpclett.1c01847. 34283619

[ref55] Mandal A.; Li X.; Huo P. Theory of vibrational polariton chemistry in the collective coupling regime. J. Chem. Phys. 2022, 156, 014101 10.1063/5.0074106. 34998324

[ref56] Weight B. M.; Krauss T.; Huo P. Investigating Molecular Exciton Polaritons Using Ab Initio Cavity Quantum Electrodynamics. J. Phys. Chem. Lett. 2023, 14, 5901–5913. 10.1021/acs.jpclett.3c01294. 37343178 PMC10316409

[ref57] Li T. E.; Hammes-Schiffer S. Electronic Born-Oppenheimer approximation in nuclear-electronic orbital dynamics. J. Chem. Phys. 2023, 158, 114118 10.1063/5.0142007. 36948810

[ref58] Haugland T. S.; Schäfer C.; Ronca E.; Rubio A.; Koch H. Intermolecular interactions in optical cavities: An ab initio QED study. J. Chem. Phys. 2021, 154, 094113 10.1063/5.0039256. 33685159

[ref59] Riso R. R.; Haugland T. S.; Ronca E.; Koch H. On the characteristic features of ionization in QED environments. J. Chem. Phys. 2022, 156, 234103 10.1063/5.0091119. 35732519

[ref60] Liebenthal M. D.; Vu N.; DePrince A. E. Equation-of-motion cavity quantum electrodynamics coupled-cluster theory for electron attachment. J. Chem. Phys. 2022, 156, 054105 10.1063/5.0078795. 35135288

[ref61] Flick J. Simple Exchange-Correlation Energy Functionals for Strongly Coupled Light-Matter Systems Based on the Fluctuation-Dissipation Theorem. Phys. Rev. Lett. 2022, 129, 143201 10.1103/PhysRevLett.129.143201. 36240406

[ref62] Flick J.; Appel H.; Ruggenthaler M.; Rubio A. Cavity Born–Oppenheimer Approximation for Correlated Electron–Nuclear-Photon Systems. J. Chem. Theory Comput. 2017, 13, 1616–1625. 10.1021/acs.jctc.6b01126. 28277664 PMC5390309

[ref63] Buchholz F.; Theophilou I.; Giesbertz K. J. H.; Ruggenthaler M.; Rubio A. Light-Matter Hybrid-Orbital-Based First-Principles Methods: The Influence of Polariton Statistics. J. Chem. Theory Comput. 2020, 16, 5601–5620. 10.1021/acs.jctc.0c00469. 32692551 PMC7482321

[ref64] McTague J.; Foley J. J. Non-Hermitian cavity quantum electrodynamics-configuration interaction singles approach for polaritonic structure with ab initio molecular Hamiltonians. J. Chem. Phys. 2022, 156, 154103 10.1063/5.0091953. 35459324

[ref65] Liebenthal M. D.; Vu N.; DePrince A. E. Mean-Field Cavity Effects in Quantum Electrodynamics Density Functional and Coupled-Cluster Theories. 2023, arXiv:2303.10821. arXiv.org e-Print archive. http://arxiv.org/abs/physics/2303.10821. 10.1021/acs.jpca.3c01842 37289181

[ref66] Vu N.; McLeod G. M.; Hanson K.; DePrince A. E. Enhanced Diastereocontrol via Strong Light-Matter Interactions in an Optical Cavity. J. Phys. Chem. A 2022, 126, 9303–9312. 10.1021/acs.jpca.2c07134. 36472381

[ref67] Philbin J. P.; Haugland T. S.; Ghosh T. K.; Ronca E.; Chen M.; Narang P.; Koch H. Molecular van der Waals Fluids in Cavity Quantum Electrodynamics. 2022, arXiv:2209.07956. arXiv.org e-Print archive. http://arxiv.org/abs/physics/2209.07956. 10.1021/acs.jpclett.3c01790 PMC10578074 37774379

[ref68] Riso R. R.; Grazioli L.; Ronca E.; Giovannini T.; Koch H. Strong Coupling in Chiral Cavities: Nonperturbative Framework for Enantiomer Discrimination. 2022, arXiv:2209.01987. arXiv.org e-Print archive. http://arxiv.org/abs/physics/2209.01987.

[ref69] Pavošević F.; Smith R. L.; Rubio A. Computational study on the catalytic control of endo/exo Diels-Alder reactions by cavity quantum vacuum fluctuations. Nat. Commun. 2023, 14, 2766 10.1038/s41467-023-38474-w. 37179341 PMC10183045

[ref70] Rokaj V.; Welakuh D. M.; Ruggenthaler M.; Rubio A. Light-Matter Interaction in the Longwavelength Limit: No Ground-State Without Dipole Self-Energy. J. Phys. B: At., Mol. Opt. Phys. 2018, 51, 034005 10.1088/1361-6455/aa9c99.

[ref71] Schäfer C.; Ruggenthaler M.; Rubio A. Ab initio nonrelativistic quantum electrodynamics: Bridging quantum chemistry and quantum optics from weak to strong coupling. Phys. Rev. A 2018, 98, 043801 10.1103/PhysRevA.98.043801.

[ref72] Vendrell O. Coherent Dynamics in Cavity Femtochemistry: Application of the Multi-Configuration Time-Dependent Hartree Method. Chem. Phys. 2018, 509, 55 10.1016/j.chemphys.2018.02.008.

[ref73] Mandal A.; Krauss T. D.; Huo P. Polariton-Mediated Electron Transfer via Cavity Quantum Electrodynamics. J. Phys. Chem. B 2020, 124, 6321–6340. 10.1021/acs.jpcb.0c03227. 32589846

[ref74] Power E. A.; Zienau S. Coulomb gauge in non-relativistic quantum electro-dynamics and the shape of spectral lines. Philos. Trans. R. Soc., A 1959, 251, 427–454.

[ref75] Cohen-Tannoudji C.; Dupont-Roc J.; Grynberg G. Photons and Atoms: Introduction to Quantum Electrodynamics; John Wiley & Sons, Inc., 1989.

[ref76] Mondal M.; Semenov A.; Ochoa M. A.; Nitzan A. Strong Coupling in Infrared Plasmonic Cavities. J. Phys. Chem. Lett. 2022, 13, 9673–9678. 10.1021/acs.jpclett.2c02304. 36215723

[ref77] Pavošević F.; Flick J. Polaritonic Unitary Coupled Cluster for Quantum Computations. J. Phys. Chem. Lett. 2021, 12, 9100–9107. 10.1021/acs.jpclett.1c02659. 34520211

[ref78] De Liberato S. Light-Matter Decoupling in the Deep Strong Coupling Regime: The Breakdown of the Purcell Effect. Phys. Rev. Lett. 2014, 112, 016401 10.1103/PhysRevLett.112.016401. 24483911

[ref79] Frisk Kockum A.; Miranowicz A.; Liberato S. D.; Savasta S.; Nori F. Ultrastrong coupling between light and matter. Nat. Rev. Phys. 2019, 1, 19–40. 10.1038/s42254-018-0006-2.

[ref80] Jaynes E. T.; Cummings F. W. Comparison of quantum and semiclassical radiation theories with application to the beam maser. Proc. IEEE 1963, 51, 89–109. 10.1109/PROC.1963.1664.

[ref81] Irish E. K.; Gea-Banacloche J.; Martin I.; Schwab K. C. Dynamics of a two-level system strongly coupled to a high-frequency quantum oscillator. Phys. Rev. B 2005, 72, 195410 10.1103/PhysRevB.72.195410.

[ref82] Irish E. K. Generalized Rotating-Wave Approximation for Arbitrarily Large Coupling. Phys. Rev. Lett. 2007, 99, 173601 10.1103/PhysRevLett.99.173601. 17995329

[ref83] Schweber S. On the application of Bargmann Hilbert spaces to dynamical problems. Ann. Phys. 1967, 41, 205–229. 10.1016/0003-4916(67)90234-5.

[ref84] Agarwal S.; Rafsanjani S. M. H.; Eberly J. H. Tavis-Cummings model beyond the rotating wave approximation: Quasidegenerate qubits. Phys. Rev. A 2012, 85, 043815 10.1103/PhysRevA.85.043815.

[ref85] Marston C. C.; Balint-Kurti G. G. The Fourier grid Hamiltonian method for bound state eigenvalues and eigenfunctions. J. Chem. Phys. 1989, 91, 3571–3576. 10.1063/1.456888.

[ref86] Tannor D. J. Introduction to Quantum Mechanics: A Time-Dependent Perspective; University Science Books: Mill Valley, USA, 2007.

[ref87] Wang D. S.; Yelin S. F. A Roadmap Toward the Theory of Vibrational Polariton Chemistry. ACS Photonics 2021, 8, 2818–2826. 10.1021/acsphotonics.1c01028.

[ref88] Chikkaraddy R.; de Nijs B.; Benz F.; Barrow S. J.; Scherman O. A.; Rosta E.; Demetriadou A.; Fox P.; Hess O.; Baumberg J. J. Single-molecule strong coupling at room temperature in plasmonic nanocavities. Nature 2016, 535, 127–130. 10.1038/nature17974. 27296227 PMC4947385

[ref89] Irish E.; Armour A. Defining the Semiclassical Limit of the Quantum Rabi Hamiltonian. Phys. Rev. Lett. 2022, 129, 183603 10.1103/PhysRevLett.129.183603. 36374670

[ref90] Li J.; Eckstein M. Manipulating Intertwined Orders in Solids with Quantum Light. Phys. Rev. Lett. 2020, 125, 217402 10.1103/PhysRevLett.125.217402. 33275019

[ref91] Pavosevic F.; Rubio A. Wavefunction embedding for molecular polaritons. J. Chem. Phys. 2022, 157, 094101 10.1063/5.0095552. 36075718

[ref92] Lindoy L. P.; Mandal A.; Reichman D. R. Quantum dynamical effects of vibrational strong coupling in chemical reactivity. Nat. Commun. 2023, 14, 2733 10.1038/s41467-023-38368-x. 37173299 PMC10182063

[ref93] Di Stefano O.; Settineri A.; Macrì V.; Garziano L.; Stassi R.; Savasta S.; Nori F. Resolution of gauge ambiguities in ultrastrong-coupling cavity quantum electrodynamics. Nat. Phys. 2019, 15, 803–808. 10.1038/s41567-019-0534-4.

[ref94] Göppert-Mayer M. Elementary Processes with Two Quantum Transitions. Ann. Phys. 2009, 521, 466–479. 10.1002/andp.200952107-804.

[ref95] Li T. E.; Chen H.-T.; Nitzan A.; Subotnik J. E. Quasiclassical modeling of cavity quantum electrodynamics. Phys. Rev. A 2020, 101, 033831 10.1103/PhysRevA.101.033831.

[ref96] Hoffmann N. M.; Schäfer C.; Säkkinen N.; Rubio A.; Appel H.; Kelly A. Benchmarking semiclassical and perturbative methods for real-time simulations of cavity-bound emission and interference. J. Chem. Phys. 2019, 151, 244113 10.1063/1.5128076. 31893926

